# Cell-free therapy with the secretome of adipose tissue-derived stem cells in rats’ frozen-thawed ovarian grafts

**DOI:** 10.1186/s13287-018-1054-3

**Published:** 2018-11-21

**Authors:** Luciana Lamarão Damous, Ana Elisa Teófilo Saturi de Carvalho, Juliana Sanajotti Nakamuta, Marcos Eiji Shiroma, Andressa Cristina Sposato Louzada, José Maria Soares-Jr, José Eduardo Krieger, Edmund C. Baracat

**Affiliations:** 1 Disciplina de Ginecologia, Laboratório de Biologia Estrutural e Molecular (LIM58), Faculdade de Medicina da Universidade de São Paulo, Dr Arnaldo av 455, 4nf floor, room 4119, Pacaembu, São Paulo 01246-903 Brazil; 2 Laboratory of Genetics and Molecular Cardiology, Heart Institute (Incor), Faculdade de Medicina da Universidade de São Paulo, Dr Enéas de Carvalho Aguiar Av 44, 10th floor, Cerqueira Cesar, São Paulo 05403-000 Brazil; 3 Baturite St, 120. Ap 91., Aclimação, São Paulo 01530-030 Brazil

**Keywords:** Fertility preservation, Ovarian transplantation, Stem cells, Secretome, Rat

## Abstract

The use of secretome may be a new strand of cell therapy, which is equal to or even superior to the injection of live cells, called cell-free therapy. In ovarian transplantation, this approach may be a therapeutic possibility for the ovarian graft in hypoxia. We designed the present study to evaluate whether the cell-free therapy with the secretome of adipose tissue-derived stem cells (ASCs) in rat frozen-thawed ovarian grafts could protect a graft against ischemic injury. A single dose of rat ASCs secretome or vehicle was injected into the bilateral frozen-thawed ovaries of 18 adult female rats immediately after an autologous transplant. Nine animals were used to control the cryopreservation protocol and were evaluated before and after the cryopreservation process. Daily vaginal smears were performed for estrous cycle evaluation until euthanasia on postoperative day 30. Follicle viability by trypan blue, graft morphology by HE, and apoptosis by TUNEL and cleaved-caspase-3 were assessed. No differences were found with respect to estrous cycle resumption and follicle viability (*p* > 0.05). However, compared with the vehicle-treated grafts, the morphology of the secretome-treated grafts was impaired, showing reduced follicular population and increased apoptosis (*p* < 0.05). ASC secretome impaired the rat frozen-thawed ovarian graft from ischemic injury. However, more studies are needed to evaluate the factors involved and the possibility of applying the secretome in scaffolds to optimize its use.

**Electronic supplementary material:**

The online version of this article (10.1186/s13287-018-1054-3) contains supplementary material, which is available to authorized users.

## Background

The techniques of ovarian transplantation and cryopreservation have significantly progressed and have become applicable in humans over the last few years. For women who have hormone-dependent malignances, ovarian tissue cryopreservation may be the only option for fertility preservation. Although these techniques are still considered to be experimental and clinical experience is limited, there have been tens of babies produced through fresh or cryopreserved ovarian grafts. These results are encouraging, but there are still insufficient data for their use in clinical practice, due to the limited number of eligible patients and standardization problems in ovarian tissue cryopreservation techniques compared to the other already-established fertility preservation techniques described in the literature. Therefore, ovarian transplantation remains an experimental technique requiring further studies [1–3].

Despite the re-establishment of the endocrine function after ovarian transplantation, the follicular depletion caused by ischemic injury is a main concern, as it is directly related to short-term graft longevity [1]. The preliminary results are encouraging, but more and larger follow-ups are needed. In the coming years, new challenges should focus on improving freezing techniques and enhancing the vascular bed before reimplantation [4]. One treatment option that may increase angiogenesis in the transplant bed is stem cell therapy.

The therapeutic potential of adipose tissue-derived stem cells (ASCs) has been demonstrated in numerous pre-clinical models, and ASC-based therapy is being evaluated in clinics with promising results. ASCs produce a large amount of secreted factors, such as cytokines, chemokines, or growth factors, which mediate diverse functions via a crosstalk between different cell types. Indeed, in case of injury, ASCs attenuate tissue damage, inhibit fibrotic remodeling and apoptosis, promote angiogenesis, stimulate endogenous stem cell recruitment and proliferation, and reduce immune responses [5–8]. This treatment seems to be interesting for reducing the ovarian graft rejection.

In an initial study of our line of research, we tested the feasibility and safety of transgenic rat ASCs for green fluorescent protein (GFP) direct injection in fresh-grafted ovaries [9]. However, the results were different in the frozen-thawed grafts, wherein the treatment induced atrophy and increased apoptosis [10]. Other studies demonstrated that the death of transplanted cells and consequential immune reactions add burdens to the host tissue, which has already been compromised by cellular debris [11].

The autocrine/paracrine factors secreted by ASCs, called the secretome, support regenerative processes in the damaged tissue, induce angiogenesis, protect cells from apoptotic cell death, and modulate immune system. It was observed that the transplanted ASCs did not necessarily engraft and differentiate and might exert their therapeutic effects through secreted trophic signals. Research in the last decade strongly suggests that ASCs-mediated benefits are closely related with their secretome. The use of the secretome may be a new strand of cell therapy, which is equal to or even superior to the injection of live cells, called cell-free therapy. This approach would have an advantage over the use of cells, and greater safety and efficacy, since it would not depend on the immuno-compatibility, in addition to being more precise, using doses that can minimize the individual biological varieties [5, 7, 12, 13].

In translational research, several paracrine effects of the secretome have already been reported, such as anti-inflammation effect, anti-apoptosis, remodeling of extracellular matrix, activation of skin stem cells, and angiogenesis [13, 14]. Recently, damaged ovaries were evaluated after transplantation of bone marrow stromal cells (BMSC)-secretome instead of the cells. These results suggested that stem cells’ secretome is expected to overcome the limitations of stem cell transplantation and becomes the basis of a novel therapy for ovarian damage [15]. However, more studies are needed to evaluate whether the secretome may be beneficial to the ovarian tissue, because there are reports of harmful effects, such as triggering a paracrine mechanism of premature senescence in young cells [16] or impairing or fostering cancer growth [17]. These data put in doubt the benefit of the secretome, and there is a need for further studies.

We designed the present study to evaluate whether the cell-free therapy with ASCs secretome in rat frozen-thawed ovarian grafts could protect the graft against the ischemic injury.

## Materials and methods

The study was carried out at Laboratory of Structural and Molecular Gynecology (LIM-58), Gynecology Discipline, Department of Obstetrics and Gynecology, Faculdade de Medicina da Universidade de Sao Paulo (FMUSP), with cooperation of Laboratory of Genetics and Molecular Cardiology/Heart Institute/FMUSP. The experimental procedures followed institutional guidelines for care and use of laboratory animals and were approved by the Ethics Committee/FMUSP (protocol 190/10).

The study sample consisted of 27 twelve-week-old adult female Wistar (*Rattus norvegicus albinus*) rats. The animals had access to a breed-specific food formula and water ad libitum throughout the experiment and were kept under adequate sanitary, lighting (12/12 h), and temperature conditions in the animal laboratory.

Nine animals were used to control cryopreservation (first assay), and the others were distributed into two study groups with nine animals each: the control group (vehicle) and the experiment group (secretome) (second assay). In the first assay, we evaluate follicular viability by trypan blue, histomorphology by HE, and immunohischemistry for apoptosis and gene profile for apoptosis by quantitative PCR. In the second assay, we evaluate histomorphology by HE and immunohischemistry for apoptosis.

### ASCs isolation and ex vivo expansion

Inguinal subcutaneous adipose tissue was collected under sterile conditions from 10-week-old male Wistar rats and rinsed with phosphate-buffered saline (PBS). ASCs were isolated, characterized, and maintained in culture as previously described [18]. In brief, harvested tissue was dissociated by digestion with 0.075% type IA collagenase (Sigma-Aldrich, Inc.) for 45 min. Enzyme activity was stopped, and the cell suspension was centrifuged at 300*g* for 15 min. Pelleted cells were recovered and plated onto 10-cm culture plates (NUNC, Rochester, NY). At 24-h intervals, cultures were washed with PBS to remove contaminating erythrocytes and other unattached cells and then reefed with fresh medium. Plating and expansion medium consisted of Dulbecco’s modified Eagle’s medium (DMEM) low glucose with 10% fetal bovine serum (FBS) and penicillin/streptomycin antibiotics (Invitrogen Corporation, Carlsbad, CA).

Cells were maintained at 37 °C with 5% CO_2_ in tissue culture dishes and fed twice a week until they reached 80% of confluence—usually within 5 to 7 days after the initial plating. Once 80% confluence was reached (passage 0), adherent cells were detached with 0.25% trypsin-EDTA (Vitrocel Embriolife, Campinas, SP, Brazil) and either replated at 1 × 10^4^ cells/cm^2^ or used for experimental procedures until passage 3.

### Secretome achievement

ASCs at passage 3 were submitted to starvation by replacing standard culture medium for medium with 0.5% of fetal bovine serum (FBS) for 18 h. After the cells were maintained with serum and phenol-free medium for 24 h, the medium rich in factors secreted by ASCs (secretome) was used as treatment of ovarian transplantation. Total protein was quantified by spectrophotometry (ND100 NanoDrop®, Thermo Fisher Scientific Inc., Co.). According to the relative amount of total protein secreted by 5 × 10^4^ cells, injections of 25 μl of secretome/ovary in rats were performed. The standardization of dose and volume to be injected were reported in previous studies [10].

### Vaginal smear collection

Before the experiment, vaginal smears were obtained daily. Only those animals showing at least two consecutive normal 4- to 5-day vaginal estrous cycles were included in the experiment. Two investigators blinded to the experimental treatments performed this analysis (LLD and MES). In case of doubt or discordant analysis, a third investigator (JMS) was requested. Based on these criteria, three animals out of 18 were excluded.

The vaginal smear was obtained with a swab soaked in physiological solution and placed on a standard slide and immediately fixed in absolute alcohol for staining using the Shorr-Harris technique. The slides were analyzed under a light microscope at × 10 and × 40 magnification. Based on the proportion of cells found in the smears, the estrous cycle phases were characterized as follows: (1) proestrus, predominance of nucleated epithelial cells; (2) estrus, predominance of anucleated, keratinized cells; and (3) diestrus, the same proportion of leukocytes and nucleated, keratinized epithelial cells.

The ovarian transplant was performed during the diestrous phase. Beginning on postoperative (PO) day 4, vaginal smears were obtained daily from each rat between 8:00 a.m. and 10:00 a.m. every day until euthanasia, which was performed between day 30 and day 35, with the rats always in diestrus.

### Collection of ovarian tissue (oophorectomy)

Wistar female rats were anesthetized intraperitoneally with xylazine and ketamine at a dose of 15 mg kg^−1^ and 60 mg kg^−1^ of body weight, respectively. After the opening of the abdominopelvic cavity, the ovaries were identified and their pedicles were clamped and immediately ligated with 4-0 nylon suture. The fallopian tubes were resected with the periovarian adipose tissue fragments. The ovaries were placed into cryovials until the cryopreservation is performed. The wall closure was performed with a 5-0 nylon monofilament thread on two planes, the peritoneum-aponeurotic muscle and the skin.

### Ovarian cryopreservation

After bilateral oophorectomy, the fresh ovary was immediately frozen in a slow cooling freezer. The whole ovaries were placed in 1.2-ml cryovials (Sigma-Aldrich®, Inc.) with M2 medium with HEPES without penicillin and streptomycin (M2-Sigma-Aldrich®, Inc.) and dimethyl sulfoxide (DMSO) (Sigma-Aldrich®, Inc.) 1.4 M as cryoprotector and held at room temperature for 5 min. The cryovials were sealed by twisting their caps, placed in a temperature-programmed freezer (CL-8800, *Cryogenesis software*, *Freezer Control*) and cooled from 25 to 10 °C at 1 °C/min, then at a rate of 0.5 °C/min to − 7 °C, and maintained at − 7 °C for 5 min. Ice nucleation was induced manually using pre-cooled forceps, and the temperature was held at − 7 °C for a further 5 min for release of latent heat fusion. The tissue was frozen at − 55 °C at a rate of 0.5 °C/min, plugged in liquid nitrogen at − 196 °C, and stored for 24 h [19].

For thawing, the cryovials were removed from liquid nitrogen and held at room temperature until the ice melted. The ovaries were washed two times for 5 min in fresh M2 medium, gently shaken to remove cryoprotectant before further processing. The ovaries were maintained in M2 at room temperature until the transplant [19].

We used nine animals for cryopreservation control, in which we evaluated the follicular viability two times: (1) in fresh tissue after oophorectomy and (2) in frozen-thawed tissue, before transplantation. In these samples, we analyzed histomorphometry (viable ovarian follicle count), follicular viability by trypan blue, and immunohistochemistry for apoptosis by cleaved-caspase-3 and TUNEL, and quantitative gene expression (qPCR) for apoptotic gene profile using real-time PCR.

### Ovarian transplantation and cell-free therapy

A second laparotomy was performed utilizing the same technique previously described. Each animal received a pair of autologous ovary transplants. With a simple stitch of 4-0 nylon suture, intact whole ovaries were implanted in the retroperitoneum in the proximity aorta and vena cava, without vascular anastomosis, each on one side of the psoas muscle.

At this point, rats were randomized into two experimental groups, according to treatment with vehicle or secretome (*n* = 7 and *n* = 8, respectively). The treatment was injected into both ovarian grafts with only one shot into the center of the ovarian parenchyma. DMEM low glucose was used as vehicle. No leakage was visually observed after injection. The procedures were carried out with the aid of a surgical microscope (× 16). The wall closure was performed with a 5-0 nylon monofilament thread on two planes, the peritoneum-aponeurotic muscle and the skin.

### Graft retrieval and histological preparation

Between PO day 30 and 35, the animals underwent a third surgical procedure, always in the diestrus phase. Once the abdominal cavity was open, the ovaries were macroscopically identified and assessed, and so was the muscle bed for its vascularization and surrounding adhesions. The grafts were subsequently removed whole.

One ovarian graft was immediately fixed in 4% paraformaldehyde for at least 24 h. After fixation, the ovaries were dehydrated, paraffin-embedded, serially sectioned at 5 μm, and mounted on glass microscope slides. Routine hematoxylin and eosin (HE) staining was performed for histologic examination with light microscopy. Three other animals were excluded due to technical problems during processing (one of vehicle group and two of secretome group).

The other ovarian graft was prepared for follicular viability assay. After this procedure, the animals were euthanized with a lethal dose of the previously used anesthetics.

### Morphological and morphometric analyses

Morphological evaluation was achieved through descriptive analyses of the grafts. Assessment of follicular quality was based on cell density, the presence or absence of pyknotic bodies, and the integrity of the basement membrane and of the oocyte. According to these criteria, follicles were classified as normal or degenerated; only the former were characterized and quantified [10].

The viable follicles were classified as follows: (1) primordial follicle, exhibiting only an oocyte and a layer of squamous cells; (2) primary follicle, exhibiting an oocyte and a layer or more of cuboidal or prismatic cells but no antrum; and (3) secondary follicle, exhibiting an oocyte and an antrum. The mature follicle was that which contained an oocyte with a voluminous antrum. The corpus luteum was that which had intact luteal cells containing a voluminous nucleus and surrounded by capillary blood vessels.

The ischemic injury was assessed as standardized in previous studies through the morphological analysis of findings such as monomorphonuclear inflammatory infiltrate, neutrophilic exudate, ischemic alterations (tissue necrosis), and congestion (vascular obliteration) [20, 21].

### Follicular viability assay

Using a protocol previously described [10], the analysis of follicle viability was carried out. A threefold manual count of each well was performed under an inverted microscope (EVOS® XL Core Cell Imaging System, AMG) at × 200 magnification to allow for individualization of the white (viable) and blue (atretic) follicles in absolute numbers as well as in percentages of viable follicles.

### Immunohistochemical apoptosis assay

Sections containing ovarian stroma were immunostained to measure apoptosis via cleaved-caspase-3 expression (SANT-SC-1226, 1:100, Santa Cruz Biotechnology, Inc. Santa Cruz, CA, EUA) and terminal deoxynucleotidyl transferase (TdT)–mediated dUTP nickend labeling (TUNEL) assay using a commercially available kit (In Situ Cell Death Detection Kit, Fluorescein, Roche, Berlin, Germany, 11684795910) following the manufacturer’s instructions. For the negative controls, the primary antibody was omitted to avoid bias.

Images of the sections were obtained using an image acquisition software system (Leica DM2500); measurements were made using the Leica QWin V3 software. Red-brown coloring of the cell cytoplasm/nucleus of the cells was specified as positive staining (any other coloring was considered negative staining). A positive cell staining assessment was performed in four different fields per animal at × 200 magnification, and the results are expressed as a percentage of the positive area (arbitrary unity/mm^2^). Two investigators blinded to the experimental treatments performed all measurements (LLD and MES). In case of doubt or discordant analysis, a third investigator (JMS) was requested.

### Quantitative PCR

Total RNA was extracted from fresh or thawed ovaries using the QIAzol Lysis Reagent and the RNeasy Micro Kit (Qiagen, Hilden, Germany) according to the manufacturer’s instructions. The solution was treated with the RNase-Free DNase Set (Qiagen, Hilden, Germany). The total RNA obtained from each sample was quantified spectrophotometrically (ND100 NanoDrop®, Thermo Fisher Scientific Inc. Co.), and the RNA integrity was assessed by electrophoresis on a 1% agarose gel.

Total RNA (1 μg) purified from each sample was transcribed into cDNA via reverse transcription using the RT^2^ First Strand Kit (Qiagen, Hilden, Germany) according to the manufacturer’s instructions. The synthesized cDNA underwent reaction in qPCR in PCR array plates (RT^2^ Profiler PCR Arrays, cat. PARN-012Z, QIAGEN-SABiosciences Corporation, USA) and the 7500 Real-Time PCR System (Applied Biosystems, CA, USA) were used. The list of 84 genes profile analyzed are detailed in Additional file 1 (Genes Profile). The qPCR results were calculated using the ΔΔCT method and specific SABiosciences software. The gene expression results are provided as fold changes, relative to the reference group (fresh ovary). The software assigns for this group (fresh ovary) a value of 1, due to ΔΔCT relative expression analysis method [22]. So, none value of fold expression lower than 1 might be considered significant.

Genes with fold regulation > 2 were considered upregulated and those with values < 2 were considered downregulated. The values were obtained for statistical analyses, but relative expression reflects the number of times that a specific gene was expressed comparing to a reference sample or group and a significant value was considered to be a threefold change in relative to the fresh ovary. All gene expression levels were normalized using the average of the housekeeping genes (Actb, B2m, Hprt1, Ldha, and Rplp1) following the software manufacturer’s instructions. Data were analyzed using Web Basis Data Analysis at https://www.google.com/url?sa=t&rct=j&q=&esrc=s&source=web&cd=2&cad=rja&uact=8&ved=2ahUKEwi_mPepoKTeAhUFTZAKHa9LA2IQFjABegQICRAB&url=https%3A%2F%2Fdataanalysis.sabiosciences.com%2Fpcr%2Farrayanalysis.php%3Fwuid%3D8fa7191b-bb49-409d-b896-8ce28966b04e%26logindata%3D%26customerdata%3D%26customeremail%3D%26platform%3DcustomArray%26format%3DX&usg=AOvVaw23-l6Q74gr7qsQXNr95B-L.

### Statistical analysis

According to the Shapiro-Wilk normality test for normal distribution, paired *t* test was utilized to compare groups before and after cryopreservation and unpaired *t* test was utilized to compare transplanted groups (vehicle and secretome). The results were expressed as mean ± standard deviation of mean (SD). All statistical analyses were performed using Graphpad Prism 7.0 (Graphpad Software Inc., CA, USA). *p* values lower than 0.05 were considered significant.

## Results

### Study of ovarian tissue before and after cryopreservation

**Fig. 1:**
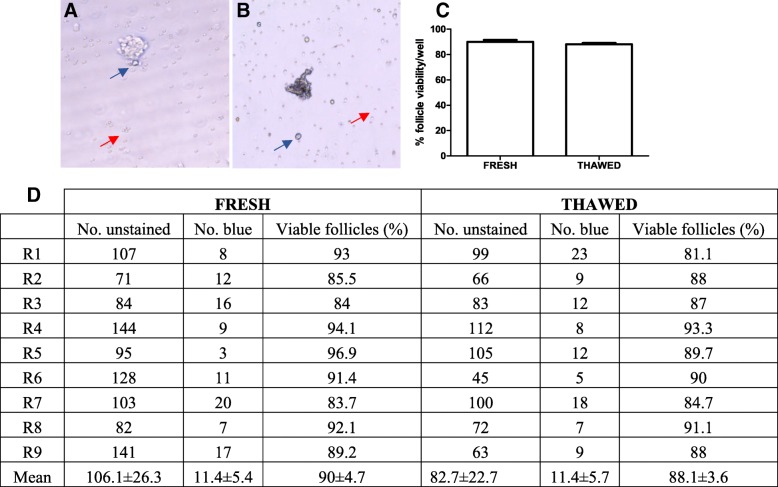
Assessment of follicular viability by trypan blue staining. Photomicrograph of viable ovarian follicles stained with white (**a**) and of nonviable follicles stained with blue (**b**). Blue arrow: trypan blue crystals. Red arrow: blood cells. × 200. Percentages (**c**) and mean number ± standard deviation (**d**) of viable ovarian follicles in fresh and thawed tissue after isolation and trypan blue staining. *p* > 0.05, paired *t* test

The ovarian follicles were easily identified, either blue or non-stained, as well as blood cells and trypan blue crystals (Fig. 1a, b). Cryopreservation did not interfere in the follicular pool, and the percentage of viable follicle loss (non-stained) was 1.9% in fresh tissue compared to cryopreserved (Fig. 1c, d).

**Fig. 2:**
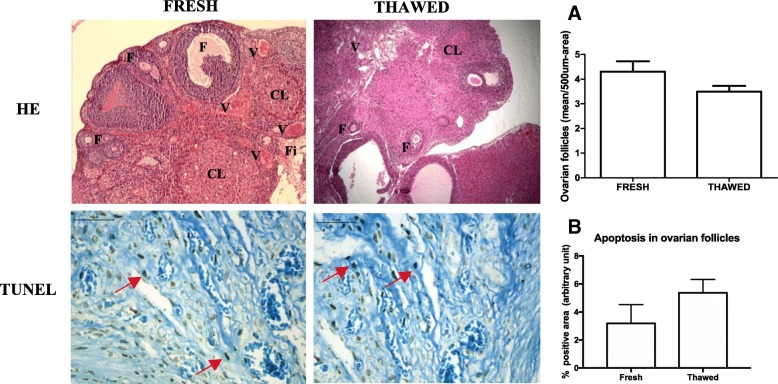
Photomicrographs of fresh and thawed ovarian tissue by HE staining and immunohistochemistry for apoptosis by TUNEL. Morphology is similar between groups. **a** Quantification of viable ovarian follicles in a 500-μm^2^ area, data are shown as the mean ± SD. Immunohistochemistry was performed on the ovarian follicles; dark brown-stained cells are considered positive (red arrow). **b** Results are expressed as a percentage of the positive area (arbitrary unity/mm^2^). Non-significant differences between groups in both analyses. *p* > 0.05, paired *t* test. HE × 100. TUNEL × 400. F follicles, CL corpora lutea, Fi fibrosis, V vessel

The morphology and morphometry of viable ovarian follicles in the fixed and stained material by HE were also similar before and after cryopreservation (fresh 4.3 ± 0.42 vs. thawed 3.5 ± 0.23, *p* > 0.05, Fig. 2). Apoptosis in the ovarian follicles was higher in the thawed ovaries than in the fresh ovaries, but these results showed no significant difference (fresh 3.24 ± 1.3 vs. thawed 5.42 ± 0.9, *p* > 0.05, Fig. 2).

**Fig. 3:**
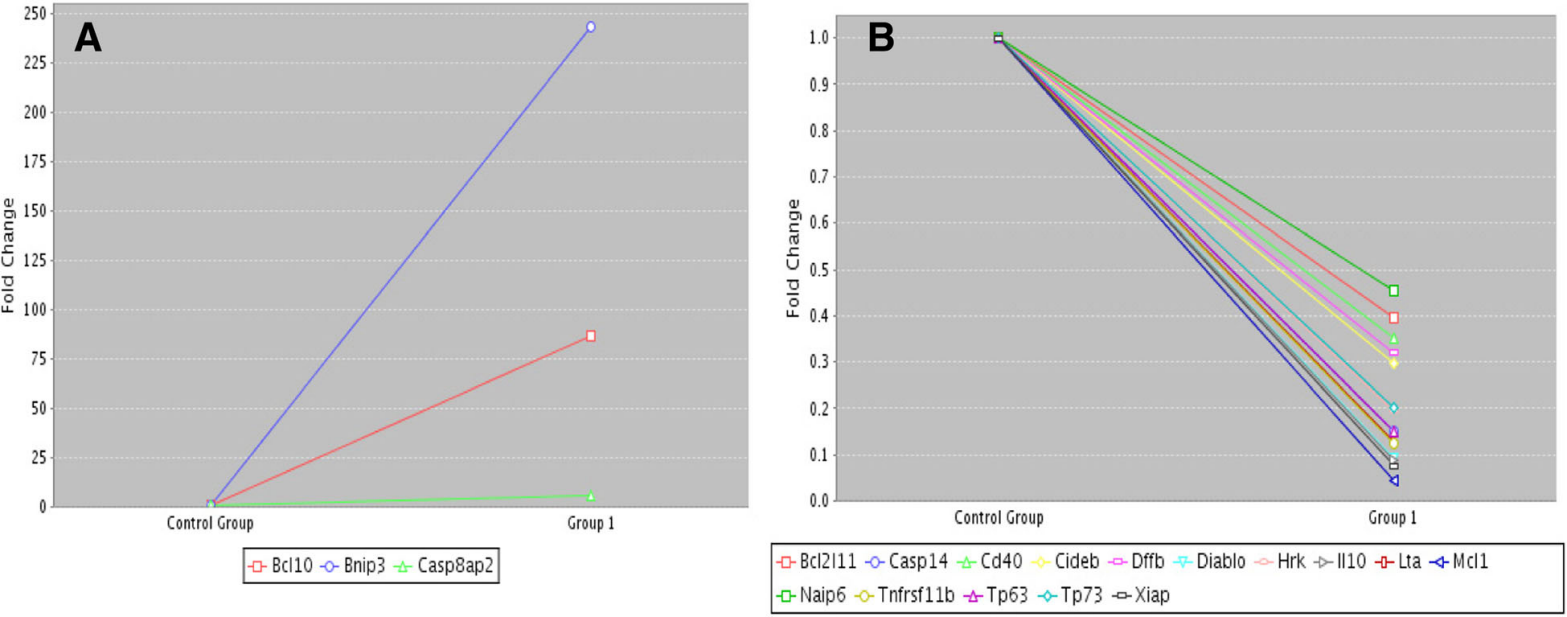
Quantitative PCR of apoptotic genes profile in fresh (control group) and thawed (group 1) ovarian tissue. **a** Upregulated genes. **b** Downregulated genes. Data were analyzed using Web Basis Data Analysis ;at https://www.google.com/url?sa=t&rct=j&q=&esrc=s&source=web&cd=2&cad=rja&uact=8&ved=2ahUKEwi_mPepoKTeAhUFTZAKHa9LA2IQFjABegQICRAB&url=https%3A%2F%2Fdataanalysis.sabiosciences.com%2Fpcr%2Farrayanalysis.php%3Fwuid%3D8fa7191b-bb49-409d-b896-8ce28966b04e%26logindata%3D%26customerdata%3D%26customeremail%3D%26platform%3DcustomArray%26format%3DX&usg=AOvVaw23-l6Q74gr7qsQXNr95B-L

The apoptotic gene expression profile showed increased expression of 3 genes (Bcl10, Bnip3, and Casp8ap2) and reduction of 15 genes (Bcl2l11, Casp14, Cd40, Cideb, Dffb, Diablo, Hrk, Il10, Lta, Mcl1, Naip6, Tnfrs11b, Tp63, Tp73, and Xiap), in according to a fold regulation values between < 2 to > 2 (Fig. 3).

### Study of the ovarian graft treated with the secretome

#### The secretome does not interfere in resumption of the estral cycle

All animals returned to cycling after the transplantation, with the day of resumption of the estrous cycle (characterized by the identification of the estrus phase in the vaginal smears) not different between treatments performed (vehicle 16.66 ± 2 vs. secretome 15.66 ± 2.88; *p* > 0.05).

#### The secretome impaired graft morphology

**Fig. 4:**
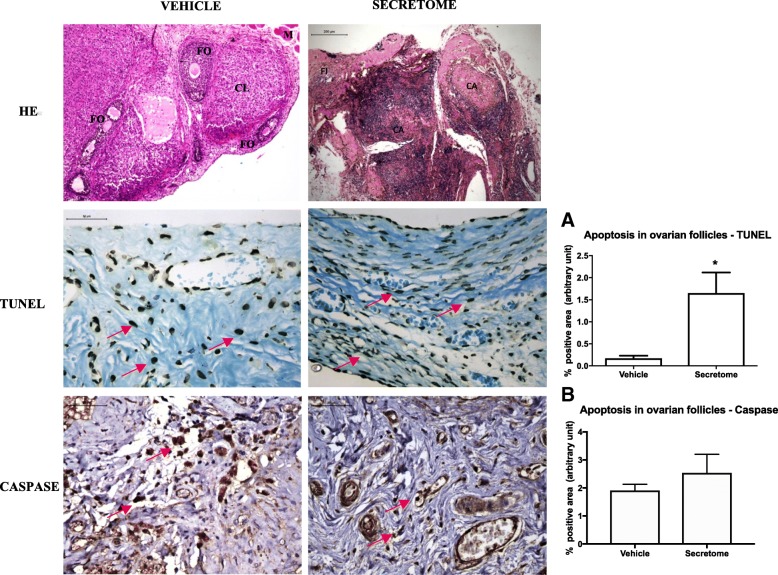
Photomicrography of cryopreserved ovarian grafts treated with vehicle or ASC secretome 30 days after an autologous avascular transplant. Treatment with secretome induced atrophy of the graft, with predominance of fibrosis and few viable follicles (HE). Immunohistochemistry for apoptosis (TUNEL and cleaved-caspase-3) was performed on the ovarian follicles, and dark brown-stained cells were considered positive (red arrows). The results are expressed as a percentage of the positive area (arbitrary unity/mm^2^). Apoptosis increased in TUNEL assay in grafts treated with the secretome (**a**) and remained similar in caspase (**b**). *p < 0.05, unpaired *t* test. HE × 50. Immunohistochemistry × 400. ASC adipose tissue-derived stem cells, CL corpora lutea, CA corpora albicans, FI fibrosis

In the vehicle-treated animals, the ovarian tissue had follicles at various stages of maturation, ranging from primordial to preovulatory follicles, sometimes with luteinization in the wall and presence of occasional corpora albicans and corpora lutea. In animals treated with secretome, there were few ovarian follicles at various stages of maturation, ranging from primordial to preovulatory follicles and moderate fibrosis (Fig. 4).

#### Secretome therapy reduced viable ovarian follicles and increased apoptosis by TUNEL

The treatment with the secretome reduced the percentage of viable follicles by trypan blue (vehicle 84.4 ± 4.4 vs. secretome 39.6 ± 6.8, *p* < 0.05) and increased apoptosis by TUNEL (vehicle 0.11 ± 0.18 vs. secretome 1.72 ± 2.65, *p* < 0.05 secretome), but by cleaved-caspase 3, there was no significant difference (vehicle 1.9 ± 0.73 vs. secretome 2.25 ± 0.73, *p* > 0.05) (Fig. 4).

## Discussion

The human endothelial stem cell secretome contains a number of molecules and growth factors that have been shown to be capable of protecting or regenerating different tissues, both in in vitro pre-clinical studies and in in vivo studies [7]. To study the profile of secreted factors, ASCs are cultured in vitro, and the secretome can be analyzed by various techniques, such as mass spectrometry, in combination with immunological assays [12], targeted proteomic, ELISA, or membrane-based growth factor antibody array [7, 14]. Using ELISA, basic fibroblast growth factor (bFGF), keratinocyte growth factor (KGF), and hepatocyte growth factor (HGF) were detected. Targeted proteomic approaches demonstrated that ASC secreted higher amounts of angiogenic and anti-apoptotic growth factors, such as hepatocyte growth factor (HGF), vascular endothelial growth factor (VEGF), stem cell factor (SCF), and nerve growth factor (NGF), as well as interleukin-6 (IL-6). In addition to the variations inherent in the technique employed, there is still variability according to the microenvironment in which the cell is being cultured. Accumulating evidence suggests that hypoxia enhances the paracrine activity of ASC by increasing the secretion of growth factors [7, 14]. In our study, the use of the secretome was harmful to the ovarian graft already in a state of hypoxia.

The use of mesenchymal cells extracted from adipose tissue of male rats is a standard technique and by the laboratory LCGM/Incor/FMUSP. Previous studies of the group have shown that cells harvested from males (Tc-labeled ASCs) maintain viability in vitro and when injected into the heart (with or without scaffolds) of females can be identified in tissues such as the liver, kidneys, and lungs, without prejudice of the same [18]. Among the advantages of using these cells, we can highlight its easy extraction, high productivity, low immunogenicity, homogeneity in culture, and plasticity (ability to differentiate into several mature cell lines) [18, 23, 24]. With respect to its use in transplanted ovarian tissue, an initial study in our line of research, rASC obtained from transgenic rats expressing green fluorescent protein (GFP) were injected in topic (intact) or freshly grafted ovaries. We noted that rASC-GFP^+^ were observed in similar quantities in both tissues with immunoexpression for GFP and von Willebrand factor (double-labeling immunofluorescence). This data suggests that rASC therapy in ovarian tissue could be feasible and safe [25].

The direct injection of ASCs was used to improve the trauma or quality of the ovarian graft in the state of hypoxia [10]. However, there was an exacerbation of the local inflammatory process. Among the hypotheses would be the direct action of ASCs or their secretome. Thus, this study shows that the application of the secretome of these cells in frozen-thawed ovarian graft may be involved in this inflammatory process, worsening the damage to the graft against the ischemic injury. These results suggest caution in the application of the secretome in ovarian transplantation.

We believe that the technique of obtaining the secretome is not related to these results, as the cultured ASCs were able to maintain protein secretion even in the absence of FBS (quantified by spectrophotometry). The starvation method is widely used in cell culture, which consists in the reduction or withdrawal of fetal bovine serum (FBS) as a supplement to the culture medium for use in transplants or collection of secretome [18, 23, 26]. Removal of the FBS from the conditioned culture medium is done to prevent its components from interfering with and/ or overlapping those secreted by the cells in culture, or even overestimating or modulating such secretion. With the removal of FBS from the conditioned medium, we can increase the confidence that the proteins present in the conditioned culture medium come from the cultured cells and not from external sources. Some studies have shown that ASCs grown at low FBS concentrations maintain viability in culture and have exacerbated functions related to secreted proteins [27, 28].

In this study, we evaluated the ovarian graft function indirectly through the analyses of estrous cycle phases by vaginal smear collection. In a previous study, our team evaluated the ovarian graft endocrine function through the measure of the thickness of the granulosa layer in the antral follicles and counted the number of follicular cells [29]. However, in this study we did not perform this additional measure because the analyses of morphology and apoptosis were conclusive with the graft impairment after the treatment with secretome. Although the return of the estrous cycle occurred in the animals treated with the secretome, the survival of the ovarian graft may be seriously compromised, taking into account the reduction of the follicular population and the increase of the apoptosis, which reduces the viability of the graft in the long term.

The slow cryopreservation protocol of the ovarian tissue used in this study preserved the tissue morphology but did not reduce the follicular pool or induce apoptosis. However, the increase in the expression of apoptotic genes such as Bnip3 and the reduction of other anti-apoptotic such as Mcl1 can show a tendency of increase of the apoptosis in the long term with consequent atrophy of this tissue [30, 31]. Likewise, the reduction in IL-10 expression may increase the inflammatory process [32].

A limiting factor of the present study is the lack of isolation of the factors involved in tissue damage and contained in the secretome. Using the same technique of obtaining the secretome, the profile of the secretome of human and mouse mesenchymal cells was already standardized by means of a customized kit *Multiplex Protein Array* for 81 cytokines (RayBio®) (JSN, unpublished observations). For rat cells, this standardization has not yet been possible and the tests are still ongoing.

In addition, the injection of the secretome was performed only once, and it may be necessary to test continuous applications to obtain some therapeutic effect, since there is no continuous secretion of the growth factors. The ASCs secretome contains exosomes and microvesicles, which may secrete yet unknown factors that may interfere with the biological processes of the host tissue. Thus, these compounds need to be studied in depth to generate more knowledge about the complexity of their actions for future application of cell-free therapy [33–35].

## Conclusion

ASCs secretome impaired the rat frozen-thawed ovarian graft from ischemic injury. However, more studies are needed to evaluate the factors involved and the possibility of applying the secretome in scaffolds to optimize its use.

## Additional file

Additional file 1: **Table S1.** Apoptosis genes profile - RT^2^ Profiler PCR Arrays, cat. PARN-012Z, Catalog #330231, QIAGEN- SABiosciences Corporation, USA. H01 to H05 are housekeeping genes. (DOC 159 kb)

## Abbreviations

ASCs: Adipose tissue-derived stem cells
bFGF: Basic fibroblast growth factor
BMSC: Bone marrow stromal cells
DMEM: Dulbecco’s modified Eagle’s medium
FBS: Fetal bovine serum
GFP: Green fluorescent protein
HE: Hematoxylin and eosin
HGF: Hepatocyte growth factor
IL-10: Interleukin-10
IL-6: Interleukin-6
KGF: Keratinocyte growth factor
NGF: Nerve growth factor
PBS: Phosphate-buffered saline
PO: Postoperative
qPCR: Quantitative gene expression
SCF: Stem cell factor
SD: Standard deviation
TUNEL: Terminal deoxynucleotidyl transferase (TdT)–mediated dUTP nickend labeling
VEGF: Vascular endothelial growth factor
