# Supplementary material for: Cell-free therapy with the secretome of adipose tissue-derived stem cells in rats’ frozen-thawed ovarian grafts
Source: Stem Cell Res Ther. 2018 Nov 21;9:323. doi: 10.1186/s13287-018-1054-3 (PMC6249760; doi:10.1186/s13287-018-1054-3)
Supplement: Supplementary file 1 — Table S1. Apoptosis genes profile - RT2 Profiler PCR Arrays, cat. PARN-012Z, Catalog #330231, QIAGEN- SABiosciences Corporation, USA. H01 to H05 are housekeeping genes. (DOC 159 kb) [file 13287_2018_1054_MOESM1_ESM.doc]

Additional File 1

**Table S1 –** Apoptosis genes profile - RT2 Profiler PCR Arrays, cat. PARN-012Z, Catalog #330231, QIAGEN- SABiosciences Corporation, USA. H01 to H05 are housekeeping genes.

| **Position** | **Unigene** | **Refseq** | **Symbol** | **Description** | **Gene name** | **RT2 Catalog** |
| --- | --- | --- | --- | --- | --- | --- |
| A01 | Rn.3105 | NM_001100850 | Abl1 | C-abl oncogene 1, receptor tyrosine kinase | Abl | PPR06527A |
| A02 | Rn.203165 | NM_031356 | Aifm1 | Apoptosis-inducing factor, mitochondrion-associated 1 | Aif/Pdcd8 | PPR44012A |
| A03 | Rn.11422 | NM_033230 | Akt1 | V-akt murine thymoma viral oncogene homolog 1 | Akt | PPR45425C |
| A04 | Rn.3318 | NM_013132 | Anxa5 | Annexin A5 | Anx5/LC5 | PPR43047A |
| A05 | Rn.64522 | NM_023979 | Apaf1 | Apoptotic peptidase activating factor 1 | - | PPR06564C |
| A06 | Rn.103083 | NM_001127379 | Api5 | Apoptosis inhibitor 5 | - | PPR55009A |
| A07 | Rn.145049 | NM_001107757 | Aven | Apoptosis, caspase activation inhibitor | - | PPR47815A |
| A08 | Rn.36696 | NM_022698 | Bad | BCL2-associated agonist of cell death | - | PPR06535A |
| A09 | Rn.16320 | NM_001106647 | Bag1 | BCL2-associated athanogene | Bag-1 | PPR06492A |
| A10 | Rn.14598 | NM_053812 | Bak1 | BCL2-antagonist/killer 1 | - | PPR06503A |
| A11 | Rn.10668 | NM_017059 | Bax | Bcl2-associated X protein | - | PPR06496C |
| A12 | Rn.13007 | NM_031328 | Bcl10 | B-cell CLL/lymphoma 10 | - | PPR06500C |
| B01 | Rn.9996 | NM_016993 | Bcl2 | B-cell CLL/lymphoma 2 | Bcl-2 | PPR06577B |
| B02 | Rn.19770 | NM_133416 | Bcl2a1 | B-cell leukemia/lymphoma 2 related protein A1d | Bcl2a1d | PPR06510A |
| B03 | Rn.10323 | NM_031535 | Bcl2l1 | Bcl2-like 1 | Bcl-xl/Bcl2l/Bclx/bcl-X | PPR06491A |
| B04 | Rn.82709 | NM_022612 | Bcl2l11 | BCL2-like 11 (apoptosis facilitator) | Bim/BimL | PPR06472A |
| B05 | Rn.44267 | NM_021850 | Bcl2l2 | Bcl2-like 2 | BCL-W/BCL-WEL/BCL-WS/Bclw | PPR06543A |
| B06 | Rn.31142 | NM_022684 | Bid | BH3 interacting domain death agonist | - | PPR50307A |
| B07 | Rn.38487 | NM_053704 | Bik | BCL2-interacting killer (apoptosis-inducing) | Biklk/Blk | PPR06537A |
| B08 | Rn.205955 | NM_021752 | Birc2 | Baculoviral IAP repeat-containing 2 | Api2/rIAP1 | PPR06435A |
| B09 | Rn.64578 | NM_023987 | Birc3 | Baculoviral IAP repeat-containing 3 | Birc2/IAP1 | PPR06459A |
| B10 | Rn.54471 | NM_022274 | Birc5 | Baculoviral IAP repeat-containing 5 | AP14 | PPR06554F |
| B11 | Rn.229165 | NM_001106835 | Bnip2 | BCL2/adenovirus E1B interacting protein 2 | - | PPR06691A |
| B12 | Rn.2060 | NM_053420 | Bnip3 | BCL2/adenovirus E1B interacting protein 3 | - | PPR06513A |
| C01 | Rn.44461 | NM_017312 | Bok | BCL2-related ovarian killer | Bok-BH3 | PPR06546A |
| C02 | Rn.138066 | NM_001130554 | Card10 | Caspase recruitment domain family, member 10 | - | PPR54418A |
| C03 | Rn.37508 | NM_012762 | Casp1 | Caspase 1 | Ice/Il1bc | PPR06427A |
| C04 | Rn.81078 | NM_130422 | Casp12 | Caspase 12 | - | PPR06567A |
| C05 | Rn.228179 | NM_001191776 | Casp14 | Caspase 14 | - | PPR57599A |
| C06 | Rn.1438 | NM_022522 | Casp2 | Caspase 2 | - | PPR06502A |
| C07 | Rn.10562 | NM_012922 | Casp3 | Caspase 3 | Lice/Yama | PPR06384B |
| C08 | Rn.16195 | NM_053736 | Casp4 | Caspase 4, apoptosis-related cysteine peptidase | Casp11 | PPR06507F |
| C09 | Rn.88160 | NM_031775 | Casp6 | Caspase 6 | Mch2 | PPR06528A |
| C10 | Rn.53995 | NM_022260 | Casp7 | Caspase 7 | - | PPR06552A |
| C11 | Rn.54474 | NM_022277 | Casp8 | Caspase 8 | CASP-8 | PPR06555A |
| C12 | Rn.198715 | NM_001107921 | Casp8ap2 | Caspase 8 associated protein 2 | - | PPR46757A |
| D01 | Rn.32199 | NM_031632 | Casp9 | Caspase 9, apoptosis-related cysteine peptidase | Apaf3/Casp-9-CTD/Casp9_v1 | PPR06529A |
| D02 | Rn.25180 | NM_134360 | Cd40 | CD40 molecule, TNF receptor superfamily member 5 | Tnfrsf5 | PPR47997A |
| D03 | Rn.44218 | NM_053353 | Cd40lg | CD40 ligand | Cd40l/Tnfsf5 | PPR49715A |
| D04 | Rn.204752 | NM_057138 | Cflar | CASP8 and FADD-like apoptosis regulator | Flip | PPR06420E |
| D05 | Rn.8171 | NM_001170467 | Cidea | Cell death-inducing DFFA-like effector a | - | PPR06597A |
| D06 | Rn.204016 | NM_001108869 | Cideb | Cell death-inducing DFFA-like effector b | - | PPR06596A |
| D07 | Rn.2202 | NM_012839 | Cycs | Cytochrome c, somatic | CYCSA | PPR42696B |
| D08 | Rn.7262 | NM_138910 | Dad1 | Defender against cell death 1 | - | PPR43803A |
| D09 | Rn.23108 | NM_001107335 | Dapk1 | Death associated protein kinase 1 | - | PPR47529A |
| D10 | Rn.6514 | NM_053679 | Dffa | DNA fragmentation factor, alpha subunit | ICAD-S | PPR06548A |
| D11 | Rn.67077 | NM_053362 | Dffb | DNA fragmentation factor, beta polypeptide (caspase-activated DNase) | Cad | PPR06565A |
| D12 | Rn.9090 | NM_001008292 | Diablo | Diablo homolog (Drosophila) | Smac | PPR44271A |
| E01 | Rn.16183 | NM_152937 | Fadd | Fas (TNFRSF6)-associated via death domain | Mort1 | PPR06629A |
| E02 | Rn.106419 | NM_080895 | Faim | Fas apoptotic inhibitory molecule | - | PPR47442A |
| E03 | Rn.162521 | NM_139194 | Fas | Fas (TNF receptor superfamily, member 6) | Tnfrsf6 | PPR47870F |
| E04 | Rn.9725 | NM_012908 | Faslg | Fas ligand (TNF superfamily, member 6) | Apt1Lg1/CD95-L/Fasl/Tnfsf6 | PPR06476A |
| E05 | Rn.10250 | NM_024127 | Gadd45a | Growth arrest and DNA-damage-inducible, alpha | Ddit1/Gadd45 | PPR06489F |
| E06 | Rn.233846 | NM_057130 | Hrk | Harakiri, BCL2 interacting protein (contains only BH3 domain) | Bid3/Dp5 | PPR06399B |
| E07 | Rn.9868 | NM_012854 | Il10 | Interleukin 10 | IL10X | PPR06479A |
| E08 | Rn.160577 | NM_080769 | Lta | Lymphotoxin alpha (TNF superfamily, member 1) | Tnfb | PPR06575A |
| E09 | Rn.19329 | NM_001008315 | Ltbr | Lymphotoxin beta receptor (TNFR superfamily, member 3) | - | PPR46808A |
| E10 | Rn.34914 | NM_053842 | Mapk1 | Mitogen activated protein kinase 1 | ERK-2/ERT1/Erk2/p42-MAPK | PPR48780A |
| E11 | Rn.44266 | NM_053777 | Mapk8ip1 | Mitogen-activated protein kinase 8 interacting protein 1 | JIP1/Jip-1/Mapk8ip | PPR49725A |
| E12 | Rn.129914 | NM_021846 | Mcl1 | Myeloid cell leukemia sequence 1 | - | PPR06541D |
| F01 | Rn.92423 | XM_006223993 | Naip6 | NLR family, apoptosis inhibitory protein 6 | Birc1/Birc1a/Birc1b/Naip/Naip2 | PPR53085B |
| F02 | Rn.2411 | NM_001276711 | Nfkb1 | Nuclear factor of kappa light polypeptide gene enhancer in B-cells 1 | EBP-1/NF-kB | PPR42746A |
| F03 | Rn.86956 | NM_053516 | Nol3 | Nucleolar protein 3 (apoptosis repressor with CARD domain) | Arc | PPR57597A |
| F04 | Rn.9346 | NM_017141 | Polb | Polymerase (DNA directed), beta | - | PPR44316G |
| F05 | Rn.2511 | NM_017169 | Prdx2 | Peroxiredoxin 2 | Tdpx1 | PPR42774C |
| F06 | Rn.9757 | NM_012630 | Prlr | Prolactin receptor | RATPRLR | PPR44463B |
| F07 | Rn.7817 | NM_172322 | Pycard | PYD and CARD domain containing | Asc | PPR06566A |
| F08 | Rn.102179 | NM_001191865 | Ripk2 | Receptor-interacting serine-threonine kinase 2 | - | PPR54833A |
| F09 | Rn.41053 | NM_001012066 | Sphk2 | Sphingosine kinase 2 | - | PPR49358F |
| F10 | Rn.2275 | NM_012675 | Tnf | Tumor necrosis factor (TNF superfamily, member 2) | RATTNF/TNF-alpha/Tnfa | PPR06411F |
| F11 | Rn.105558 | NM_001108873 | Tnfrsf10b | Tumor necrosis factor receptor superfamily, member 10b | - | PPR56024B |
| F12 | Rn.202973 | NM_012870 | Tnfrsf11b | Tumor necrosis factor receptor superfamily, member 11b | Opg | PPR06478A |
| G01 | Rn.11119 | NM_013091 | Tnfrsf1a | Tumor necrosis factor receptor superfamily, member 1a | TNFR-1/Tnfr1 | PPR06395A |
| G02 | Rn.83633 | NM_130426 | Tnfrsf1b | Tumor necrosis factor receptor superfamily, member 1b | Tnfr2 | PPR06734A |
| G03 | Rn.83627 | NM_145681 | Tnfsf10 | Tumor necrosis factor (ligand) superfamily, member 10 | Trail | PPR06735B |
| G04 | Rn.3211 | NM_001001513 | Tnfsf12 | Tumor necrosis factor ligand superfamily member 12 | TWEAK | PPR43003E |
| G05 | Rn.54443 | NM_030989 | Tp53 | Tumor protein p53 | Trp53/p53 | PPR06553A |
| G06 | N/A | XM_223012 | Tp53bp2 | Tumor protein p53 binding protein, 2 | Trp53bp2 | PPR50465A |
| G07 | Rn.42907 | NM_019221 | Tp63 | Tumor protein p63 | Ket/P73l/Tp73l/Trp63 | PPR06539B |
| G08 | Rn.103860 | NM_001108696 | Tp73 | Tumor protein p73 | P73/Trp73 | PPR55284A |
| G09 | Rn.18545 | NM_001100480 | Tradd | TNFRSF1A-associated via death domain | - | PPR06738A |
| G10 | Rn.105232 | NM_001107815 | Traf2 | Tnf receptor-associated factor 2 | - | PPR55844B |
| G11 | Rn.12033 | NM_001108724 | Traf3 | Tnf receptor-associated factor 3 | - | PPR50724A |
| G12 | Rn.91239 | NM_022231 | Xiap | X-linked inhibitor of apoptosis | Api3/Birc4/riap3 | PPR06444A |
| H01 | Rn.94978 | NM_031144 | Actb | Actin, beta | Actx | PPR06570C |
| H02 | Rn.1868 | NM_012512 | B2m | Beta-2 microglobulin | - | PPR42607A |
| H03 | Rn.47 | NM_012583 | Hprt1 | Hypoxanthine phosphoribosyltransferase 1 | Hgprtase/Hprt | PPR42247F |
| H04 | Rn.107896 | NM_017025 | Ldha | Lactate dehydrogenase A | Ldh1 | PPR56603B |
| H05 | Rn.973 | NM_001007604 | Rplp1 | Ribosomal protein, large, P1 | - | PPR42363C |
